# Temporal Trends in the Association Between Female Sex and Ischemic Stroke Among Patients With Atrial Fibrillation

**DOI:** 10.1161/JAHA.124.040325

**Published:** 2025-06-23

**Authors:** Mark T. Mills, Tommaso Bucci, Peter Calvert, Dhiraj Gupta, Gregory Y. H. Lip

**Affiliations:** ^1^ Liverpool Centre for Cardiovascular Science at University of Liverpool Liverpool John Moores University and Liverpool Heart and Chest Hospital Liverpool UK; ^2^ Department of Cardiology Liverpool Heart and Chest Hospital NHS Foundation Trust Liverpool UK; ^3^ Department of Clinical Internal, Anaesthesiologic and Cardiovascular Sciences Sapienza University of Rome Rome Italy; ^4^ Danish Center for Health Services Research, Department of Clinical Medicine Aalborg University Aalborg Denmark

**Keywords:** atrial fibrillation, female sex, risk prediction, stroke, temporal trends, Atrial Fibrillation

## Abstract

**Background:**

Female sex has historically been associated with higher risk of ischemic stroke in patients with atrial fibrillation. However, contemporary European studies suggest this association may have attenuated and become nonsignificant over recent years. This study aims to characterize temporal trends in cardiovascular outcomes in a large, global cohort of patients with atrial fibrillation.

**Methods:**

Nonanticoagulated patients with newly diagnosed atrial fibrillation were identified from a global federated research network (TriNetX) between 2000 and 2019. One‐year ischemic stroke risk and risk ratios were calculated for women versus men. Secondary outcomes included all‐cause death, myocardial infarction, heart failure, and dementia. Cohorts were compared before and after adjustment for age and comorbidities.

**Results:**

Overall, 1 204 852 patients were included (44% women). Unadjusted risk of ischemic stroke increased in women (1.75% to 4.24%) and men (1.13% to 3.55%) from 2000–2004 to 2015–2019, while all‐cause death decreased over the same periods (women, 10.36% to 7.79%; males, 10.76% to 7.59%). After adjustment, female sex remained independently associated with higher risk of ischemic stroke, although the risk decreased over time (2000–2004: risk ratio, 1.54 [95% CI, 0.94–2.51]; 2015–2019: risk ratio, 1.09 [95% CI, 1.06–1.13]). After adjustment, male sex was associated with risk of all‐cause death and myocardial infarction, while risk of dementia and heart failure was similar between sexes.

**Conclusions:**

Between 2000 and 2019, the risk of ischemic stroke increased among nonanticoagulated patients with atrial fibrillation. While the association between female sex and ischemic stroke decreased over time, female sex remained associated with a higher stroke risk in 2015 to 2019 after adjustment.

## Graphical abstract

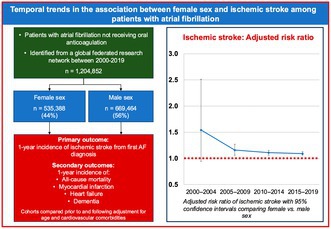

**Nonstandard Abbreviations and Acronyms**
FinACAF: Finnish Anticoagulation in Atrial Fibrillation
PSM: propensity score matching

**Research Perspective**

**What Is New?**

- In this global cohort of >1.2 million nonanticoagulated patients with atrial fibrillation, female sex remained an independent stroke risk modifier over a 20‐year period between 2000 and 2019, despite an attenuation in its impact over time.

**What Question Should Be Addressed Next?**

- Future studies should evaluate whether further declines in the association between female sex and ischemic stroke occur in diverse international atrial fibrillation populations, and how evolving health care improvements influence this trend.
- Prospective studies should assess whether modifications to current risk stratification scores, such as removing female sex as a risk modifier, improves stroke prediction and anticoagulation decision making in contemporary atrial fibrillation populations.

Atrial fibrillation (AF), the most common sustained cardiac arrhythmia, confers significant morbidity and mortality due to an increased risk of ischemic stroke, heart failure, and dementia.^1^ Numerous comorbidities contribute to stroke risk in AF, many of which are included in validated stroke risk prediction scores: heart failure, hypertension, age, diabetes, prior ischemic stroke or transient attack, and atherosclerotic vascular disease.^2^, ^3^

Historical data from the 1990s and 2000s demonstrated that female sex associates with increased risk of ischemic stroke,^4^, ^5^ although female patients with AF were generally older, with higher burdens of comorbidities than male patients.^6^ As a result, in 2010, female sex was incorporated into the CHA_2_DS_2_‐VASc score,^2^ which was found to outperform the older CHADS_2_ score,^3^ leading to its incorporation into guidelines globally.^7^, ^8^ Subsequently, data emerged to support that, in the absence of nonsex risk factors, stroke risk was similar between sexes, while in the presence of ≥1 nonsex risk factors, female sex modified and accentuated stroke risk.^9^ As such, the female sex criterion was proposed as a “risk modifier” rather than a risk factor per se.^5^

More recently, a Finnish national registry observed that the risk of ischemic stroke associated with female sex attenuated over time, becoming nonsignificant by 2018.^10^ Further, the nonsex CHA_2_DS_2_‐VASc risk score (termed CHA_2_DS_2_‐VA) demonstrated marginal superiority over CHA_2_DS_2_‐VASc in more contemporary data from this cohort, compared with a decade ago when CHA_2_DS_2_‐VASc actually performed better.^11^ Similarly, in a nationwide analysis from the United Kingdom, stroke risk prediction was comparable using CHA_2_DS_2_‐VA and CHA_2_DS_2_‐VASc risk scores.^12^ Whether these trends apply to international populations with AF remains unclear.

In the present study, we sought to explore temporal trends in ischemic stroke and cardiovascular events associated with female sex in a large, global cohort of patients with AF over a 20‐year period.

## Methods

### Data Availability Statement

The data used in this study are available on the TriNetX Research Network Database (trinetx.com; TriNetX LLC, Cambridge, MA). Access to TriNetX data requires submitting requests to TriNetX and a data‐sharing agreement.

### Study Design, Setting, and Participants

We conducted a retrospective analysis using the TriNetX Research Network Database. TriNetX is a global, federated research platform providing real‐time access to data derived from electronic health care record systems from >250 million patients at >120 global health care institutions in 19 countries in North and South America, Europe, the Middle East, Africa, and Asia‐Pacific.^13^, ^14^, ^15^, ^16^ To satisfy legal and ethical frameworks, and to prevent data reidentification, the identification of participating organizations and their contribution to each data set is not disclosed. The database includes data from inpatient and outpatient settings from academic and community‐based organizations. Participating institutions contribute deidentified, aggregated data sets, which are curated and mapped to standardized health care coding classifications and terminologies such as the *International Classification of Diseases, Tenth Edition* (*ICD‐10*), *Current Procedural Terminology*, and RxNorm.^17^, ^18^ Robust data quality control measures are overseen and implemented by TriNetX in conjunction with participating health care organizations.^19^ Prescription data for medications, including oral anticoagulants, are obtained from pharmacy claims and prescribing systems within the electronic health records. Within the online TriNetX platform, no patient identifiable information is available for analysis, and deidentified information only is presented using aggregated counts and statistical summaries. As a federated network, ethical approval for individual studies is not required. Our study is presented in accordance with the Strengthening the Reporting of Observational Studies in Epidemiology guidelines.

We identified patients aged 18–89 years with a first‐time *ICD‐10* diagnosis code of I48 (atrial fibrillation and flutter, referred to collectively as AF) from January 1, 2000, to December 31, 2019. To assess outcomes only in patients without oral anticoagulation use, those with an oral anticoagulant prescription in the 24 months before AF diagnosis were excluded, as were those with a prescription in the 12 months after diagnosis.

Baseline characteristics of patients at the time of AF diagnosis were extracted, including sex, age, body mass index, cardiovascular comorbidities forming part of the CHA_2_DS_2_‐VASc score (heart failure, hypertension, diabetes, ischemic stroke, and vascular disease [myocardial infarction, peripheral vascular disease, or aortic plaque]), history of dementia, and history of sleep apnea. Individual patient and aggregated CHA_2_DS_2_‐VASc scores are not recorded within TriNetx, and were therefore not available for analysis. *ICD‐10* codes used to identify baseline characteristics are summarized in Data S1. The cohort query design is illustrated in Table S1. The TriNetX database query was performed on July 16, 2024.

### Outcomes

The primary outcome was the risk and risk ratio (RR) of ischemic stroke within 1 year of first AF diagnosis in women versus men. Secondary outcomes included all‐cause death, myocardial infarction, acute heart failure episode, and dementia during 1‐year follow‐up. Outcomes were assessed from the date of inclusion (date of first AF diagnosis as per *ICD‐10* coding) to 365 days after inclusion or death (if before 365 days). The *ICD‐10* codes used to identify these outcomes are summarized in Data S1. To ensure a stable patient population was included, an exclusion period of 14 days after the diagnosis of AF was applied, as per previous analyses.^11^

### Statistical Analysis

Statistical analyses were performed on the TriNetX online research platform. The study period from 2000 to 2019 was divided into 5‐year intervals: January 1, 2000, to December 31, 2004; January 1, 2005, to December 31, 2009; January 1, 2010, to December 31, 2014; and January 1, 2015, to December 31, 2019. Baseline characteristics are presented as percentages or mean±SD as appropriate and were compared using χ^2^ tests for categorical variables and independent‐sample *t* tests for continuous variables. One‐year risk and RRs with 95% CIs were calculated for patients in each 5‐year interval. Analyses were performed both before and after adjustment between female and male cohorts. Adjustment was undertaken using 1:1 propensity score matching (PSM) to balance for age and cardiovascular comorbidities at index presentation (heart failure, hypertension, diabetes, ischemic stroke, myocardial infarction, peripheral vascular disease, aortic plaque, dementia, and sleep apnea). TriNetX uses a built‐in logistic regression algorithm to generate propensity scores, followed by greedy nearest neighbor matching with a caliper of 0.1 pooled SMD deviations to identify the matched subsets. *P* values <0.05 were considered significant. Unless stated otherwise, results are presented for women versus men.

## Results

### Baseline Characteristics

We identified 1 204 852 patients over the 20‐year study period (women, 535 388 [44.4%]; men, 669 464 [55.6%]): 5632 from 2000 to 2004; 120 289 from 2005 to 2009; 362 018 from 2010 to 2014; and 716 913 from 2015 to 2019. Baseline demographics during each 5‐year interval are summarized in Table 1, both before and after PSM.

**Table 1:** Baseline Demographics in Unadjusted and Adjusted Cohorts

Unadjusted cohorts																
	2000–2004				2005–2009				2010–2014				2015–2019			
	Women (2452)	Men (3180)	*P* value	SMD	Women (53492)	Men (66797)	*P* value	SMD	Women (161834)	Men (200184)	*P* value	SMD	Women (317610)	Men (399303)	*P* value	SMD
Age, y, mean±SD	69.0±14.2	64.8±14.8	<0.001	0.292	71.0±14.4	66.5±14.5	*<0.001*	0.310	71.6±14.6	67.7±14.5	*<0.001*	0.265	72.8±14.1	68.6±14.0	*<0.001*	0.262
Body mass index, mean±SD	27.7±7.3	28.0±5.7	0.530	0.043	28.0±7.6	28.2±6.2	*0.028*	0.030	28.8±7.9	28.8±6.4	0.608	0.032	29.1±8.1	29.0±6.6	*<0.001*	0.020
Hypertensive disease, %	30.9	26.4	*<0.001*	0.100	29.1	24.9	*<0.001*	0.095	31.3	27.7	*<0.001*	0.079	34.7	31.5	*<0.001*	0.070
Diabetes, %	13.4	12.0	0.010	0.044	12.3	11.7	*<0.001*	0.019	12.6	12.3	*0.034*	0.007	14.8	14.5	0.006	0.007
Heart failure, %	11.9	11.8	0.931	0.002	10.2	9.5	*<0.001*	0.025	9.4	8.8	*<0.001*	0.021	9.8	9.0	*<0.001*	0.027
Myocardial infarction, %	4.1	5.3	*0.035*	0.057	3.3	4.2	*<0.001*	0.046	2.8	3.6	*<0.001*	0.047	3.4	4.1	*<0.001*	0.034
Peripheral vascular disease, %	3.8	3.7	0.985	0.001	3.3	3.4	0.340	0.006	2.9	3.2	*<0.001*	0.016	3.8	4.0	*0.002*	0.009
Ischemic stroke, %	2.2	1.9	0.466	0.020	2.4	2.0	*<0.001*	0.030	3.2	2.7	*<0.001*	0.035	4.8	3.9	*<0.001*	0.040
Atherosclerosis of aorta, %	0.7	1.1	0.091	0.046	1.0	0.9	*0.002*	0.018	1.1	0.9	*<0.001*	0.021	1.9	1.6	*<0.001*	0.022
Alzheimer disease, %	1.6	0.7	*0.001*	0.084	1.1	0.5	*<0.001*	0.059	0.9	0.5	*<0.001*	0.052	1.2	0.6	*<0.001*	0.061
Vascular dementia, %	0.6	0.1	0.143	0.039	0.4	0.3	*<0.001*	0.030	0.3	0.2	*<0.001*	0.022	0.5	0.3	*<0.001*	0.027
Unspecified dementia, %	1.8	0.9	*0.004*	0.076	1.2	0.6	*<0.001*	0.060	1.8	1.0	*<0.001*	0.065	2.9	1.6	*<0.001*	0.084
Sleep apnea, %	1.2	1.8	0.086	0.047	1.9	2.7	*<0.001*	0.052	3.1	4.1	*<0.001*	0.057	5.0	6.3	*<0.001*	0.058

Adjusted cohorts (propensity score matched for age and cardiovascular comorbidities*)																
	2000–2004				2005–2009				2010–2014				2015–2019			
	Women (2290)	Men (2290)	*P* value	SMD	Women (48881)	Men (48881)	*P* value	SMD	Women (150227)	Men (150227)	*P* value	SMD	Women (297369)	Men (297369)	*P* value	SMD
Age, y, mean±SD	68.2±14.0	67.9±13.8	0.473	0.021	69.7±14.2	69.5±14.0	0.072	0.012	70.5±14.6	70.3±14.3	*<0.001*	0.014	71.3±14.0	71.1±13.8	*<0.001*	0.012
Body mass index, mean±SD	27.7±7.4	27.6±5.5	0.856	0.014	28.2±7.7	27.9±6.0	*0.002*	0.046	29.1±8.0	28.5±6.3	*<0.001*	0.085	29.4±8.2	28.7 ±6.5	*<0.001*	0.099
Hypertensive disease, %	28.6	27.8	0.533	0.018	26.9	27.5	*0.018*	0.015	29.1	30.0	*<0.001*	0.020	33.0	33.7	*<0.001*	0.014
Diabetes, %	12.6	12.5	0.894	0.004	12.1	12.3	0.282	0.007	12.5	12.8	*0.021*	0.009	14.6	15.2	*<0.001*	0.014
Heart failure, %	12.0	10.9	0.246	0.034	9.6	9.5	0.761	0.002	8.9	9.1	*0.032*	0.008	9.3	9.6	*<0.001*	0.010
Myocardial infarction, %	4.2	3.7	0.324	0.029	3.5	3.3	*0.023*	0.015	3.0	2.9	0.108	0.006	3.5	3.4	*0.038*	0.005
Peripheral vascular disease, %	3.7	3.6	0.874	0.005	3.3	3.1	0.143	0.009	3.0	2.9	0.163	0.005	3.9	3.8	0.475	0.002
Ischemic stroke, %	2.0	1.9	0.915	0.003	2.2	2.1	0.250	0.007	2.9	2.9	0.845	0.001	4.3	4.5	*<0.001*	0.010
Atherosclerosis of aorta, %	0.7	0.6	0.576	0.017	0.9	0.9	0.281	0.007	1.0	1.0	0.543	0.002	1.8	1.8	0.769	0.001
Alzheimer disease, %	0.8	1.0	0.638	0.014	0.6	0.7	*0.024*	0.015	0.5	0.6	*0.003*	0.011	0.7	0.8	0.001	0.008
Vascular dementia, %	0.4	0.4	>0.999	<0.001	0.3	0.3	0.291	0.007	0.3	0.3	0.458	0.003	0.4	0.4	*0.488*	0.002
Unspecified dementia, %	1.1	1.3	0.684	0.012	0.7	0.8	0.050	0.013	1.1	1.3	*<0.001*	0.016	1.9	2.1	*<0.001*	0.014
Sleep apnea, %	1.3	1.0	0.334	0.029	2.0	2.0	0.717	0.002	3.3	3.3	0.721	0.001	5.3	5.5	*0.006*	0.007

* Cardiovascular comorbidities: body mass index, heart failure, hypertensive disease, age, diabetes, stroke (ischemic), vascular disease (myocardial infarction, peripheral vascular disease, or atherosclerosis of aorta), dementia, sleep apnea.

Before PSM, female patients were generally older, with a higher prevalence of prior ischemic stroke, hypertensive disease, heart failure, diabetes, and dementia, while male patients had a higher prevalence of myocardial infarction, peripheral vascular disease, and sleep apnea. Notably, the prevalence of cardiovascular comorbidities increased during the study period in both women and men. For example, the prevalence of hypertensive disease increased from 30.9% to 34.7% in female patients between the start and end of the study, and from 26.4% to 31.5% in male patients. Age at AF diagnosis increased by 3.8 years in female patients (2000–2004: 69.0±14.2 years versus 2015–2019: 72.8±14.1 years) and by 2.8 years in male patients (64.8±14.8 years versus 68.6±14.0 years) over the study period. Similarly, body mass index increased in both female (27.7±7.3 versus 29.1 ±8.1 kg/m^2^) and male patients (28.0 ±5.7 versus 29.0±6.6 kg/m^2^) from 2000 to 2004 to 2015 to 2019.

Following PSM, female and male patients were well matched for age and cardiovascular comorbidities, with SMDs of <0.1 for all characteristics.

### Ischemic Stroke

**Figure 1:**
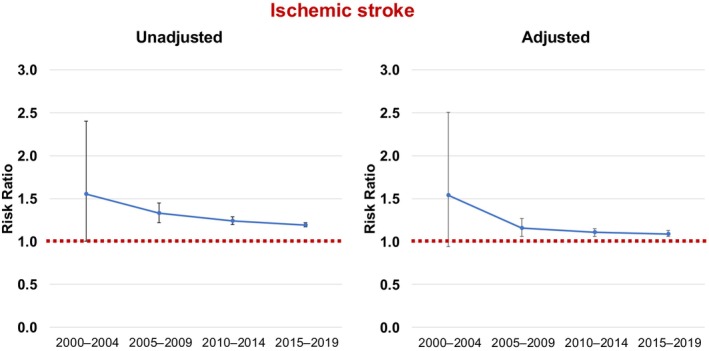
Unadjusted and adjusted risk ratios of ischemic stroke with 95% CIs, comparing women with men (red line) over time.

The 1‐year unadjusted ischemic stroke risk increased over the study period, both in women (from 1.75% in 2000–2004 to 4.24% in 2015–2019) and men (from 1.13% to 3.55%) (Table 2). Before PSM, female sex was associated with a higher risk of ischemic stroke throughout the study periods (Table 2 and Figure 1). After PSM, female sex remained independently associated with higher risk of ischemic stroke, although the RR decreased over time (2000–2004: RR, 1.54 [95% CI, 0.94–2.51]; 2005–2009: RR, 1.16 [95% CI, 1.06–1.27]; 2010–2014: RR, 1.11 [95% CI, 1.06–1.13]; 2015–2019: RR, 1.09 [95% CI, 1.06–1.13]) (Figure 1).

**Table 2:** One‐Year Outcomes in Unadjusted Cohorts, Comparing Women With Men

	Women			Men			Women vs men	
	Number at risk	Number with event	Risk, %	Number at risk	Number with event	Risk, %	Risk ratio (95% CI)	*P* value
Ischemic stroke								
2000–2004	2452	43	1.75	3180	36	1.13	1.55 (1.00–2.40)	0.049
2005–2009	53 492	1113	2.08	66 797	1044	1.56	1.33 (1.22–1.45)	<0.001
2010–2014	161 834	5445	3.37	200 184	5424	2.71	1.24 (1.20–1.29)	<0.001
2015–2019	317 610	13 457	4.24	399 303	14 164	3.55	1.19 (1.17–1.22)	<0.001
All‐cause death								
2000–2004	2452	254	10.36	3180	342	10.76	0.96 (0.83–1.12)	0.632
2005–2009	53 492	5097	9.53	66 797	6121	9.16	1.04 (1.00–1.08)	0.031
2010–2014	161 834	14 009	8.66	200 184	16 850	8.42	1.03 (1.01–1.05)	0.010
2015–2019	317 610	24 746	7.79	399 303	30 338	7.59	1.03 (1.01–1.04)	0.002
Myocardial infarction								
2000–2004	2452	56	2.28	3180	90	2.83	0.81 (0.58–1.12)	0.201
2005–2009	53 492	1036	1.94	66 797	1464	2.19	0.88 (0.82–0.96)	0.002
2010–2014	161 834	3625	2.24	200 184	5573	2.78	0.81 (0.77–0.84)	<0.001
2015–2019	317 610	8837	2.64	399 303	12 275	3.07	0.86 (0.84–0.88)	<0.001
Heart failure								
2000–2004	2452	18	0.73	3180	22	0.69	1.06 (0.57–1.97)	0.851
2005–2009	53 492	796	1.49	66 797	834	1.25	1.19 (1.08–1.31	<0.001
2010–2014	161 834	4351	2.69	200 184	5025	2.51	1.07 (1.03–1.12)	0.008
2015–2019	317 610	13 537	4.26	399 303	15 803	3.96	1.08 (1.05–1.10)	<0.001
Outcome, dementia								
2000–2004	2452	55	2.24	3180	30	0.94	2.38 (1.52–3.70)	<0.001
2005–2009	53 492	954	1.78	62 656	684	1.02	1.74 (1.58–1.92)	<0.001
2010–2014	161 834	2542	1.57	200 184	2025	1.01	1.55 (1.47–1.65)	<0.001
2015–2019	317 610	6966	2.19	399 303	5725	1.43	1.53 (1.48–1.58)	<0.001

### Secondary Outcomes

**Figure 2:**
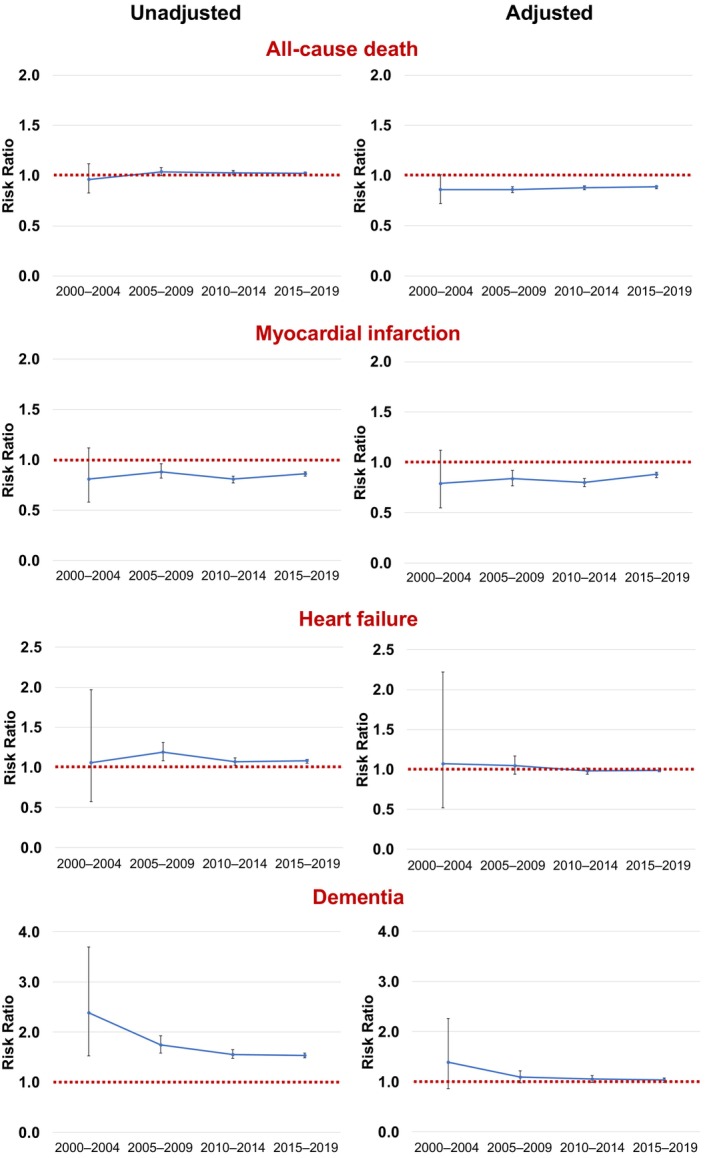
Unadjusted and adjusted risk ratios. Presented for all‐cause death, myocardial infarction, heart failure, and dementia with 95% CIs, comparing women with men (red line) over time.

The 1‐year unadjusted all‐cause mortality risk decreased over the study period, in both women (from 10.36% in 2000–2004 to 7.79% in 2015–2019) and men (10.76% to 7.59%) (Table 2). Before PSM, female sex was associated with higher risk of all‐cause death through the majority of the study period (except 2000–2004: RR, 0.96 [95% CI, 0.83–1.12]) (Table 2); however, after PSM, male sex became independently associated with higher risk of all‐cause death throughout the study period (2000–2004: RR, 0.86 [95% CI, 0.72–1.01]; 2005–2009: RR, 0.86 [95% CI, 0.83–0.89]; 2010–2014: RR, 0.88 [95% CI, 0.86–0.90]; 2015–2019: RR, 0.89 [95% CI, 0.87–0.90]) (Figure 2). Following PSM, male sex was associated with higher risk of myocardial infarction during 1‐year follow‐up (2015–2019: RR, 0.88 [95% CI, 0.85–0.90]), while risk of heart failure or dementia was similar between sexes (Figure 2).

A breakdown of patient numbers, events, risks, and RRs for all outcomes over 5‐year intervals after PSM is summarized in Table S2.

## Discussion

This study is, to the best of our knowledge, the largest to describe temporal trends in the association between female sex and cardiovascular events in a global population of nonanticoagulated patients with AF. The primary findings from our analysis are:

1. In nonanticoagulated patients with AF, the risk of ischemic stroke increased in both female and male patients over a 20‐year period from 2000 to 2019.

2. The rise in stroke risk was accompanied by an increase in cardiovascular comorbidities.

3. After adjustment for age and cardiovascular comorbidities, female sex was independently associated with higher risk of ischemic stroke throughout the 20‐year period, although this risk attenuated over time.

4. After adjustment, male sex was associated with higher risk of all‐cause death and myocardial infarction, with no difference in heart failure or dementia between sexes.

Overall, these data highlight the changing trends in AF‐related ischemic stroke over the past 2 decades and suggest that female sex may remain an important stroke risk modifier in patients with AF.

Female sex has historically been associated with a higher stroke risk in AF, likely as a result of older age at AF diagnosis in women, higher comorbidity burden, and lower anticoagulation rates.^5^, ^6^, ^20^ While the interaction between female sex and ischemic stroke risk has been extensively studied over recent decades,^5^ little is known on temporal trends in this association. The FinACAF (Finnish Anticoagulation in Atrial Fibrillation) registry is, to our knowledge, the only study to have explored this, in a population of 229 565 patients from 2007 to 2018 in Finland.^10^ Similar to the FinACAF study, our analysis demonstrates that the association between female sex and ischemic stroke risk decreased between 2000 and 2019, although unlike in FinACAF, this association still remained significant by the end of the study period. Indeed, female sex conferred a 9% higher risk of ischemic stroke in 2015 to 2019 in our analysis.

These diverging observations may be partly explained by patient selection and underlying risk profile, having included patients from global health care organizations in our study, rather than those from a single country in the FinACAF. Indeed, trends are likely to vary internationally, and Finland may be ahead of other countries in terms of reductions in sex inequalities in patients with AF.^21^ The differences in AF presentation and management between female and male patients are well described, and require the holistic education of physicians and further research to overcome.^22^ We hypothesize that, should the observed global trend in declining stroke risk associated with female sex continue over the coming decades, female sex may eventually become nonsignificantly associated with stroke risk, similar to the Finnish cohort. This observation should prompt future regular review of these trends in varying AF populations worldwide to iteratively refine stroke risk prediction scores.

The declining association between female sex and AF‐associated stroke risk is likely multifactorial, although not explicitly explainable from our data set. Generally, improvements in socioeconomic conditions have likely contributed to reductions in gender inequalities,^23^ and recent reports also suggest narrowings in general health‐related sex disparities.^24^ The inclusion of female sex into the CHA_2_DS_2_‐VASc score itself may have raised awareness of sex differences in AF‐related ischemic stroke, leading to the decline in male–female differences in AF‐related stroke rates. However, whether removal of the sex criterion (CHA_2_DS_2_‐VA) may reverse this decline over the next years remains to be seen. The increasing prevalence of cardiovascular comorbidities observed over time in our study in both female and male patients may reflect improved screening, diagnosis, and treatment of comorbidities in both sexes. Indeed, the holistic management of AF has been associated with improved clinical outcomes^25^, ^26^, ^27^ and may explain some of the reductions in these disparities.

Similarly, the association between female sex and the development of dementia decreased over time in our study in the unadjusted cohorts but remained significantly associated by end of the observation period, with a 50% to 60% higher risk in female patients. However, after adjustment, this association became marginally nonsignificant. The association between female sex and dementia risk in AF is well established,^28^, ^29^ although the reasons are not fully understood. Possible explanations include older age at AF diagnosis than men, higher cardiovascular comorbidity burden, and lower anticoagulation rates leading to a higher risk of silent cerebral infarction and cognitive impairment.^29^ Our findings highlight the need for targeted strategies to reduce these sex‐based differences in clinical practice.^30^

An important observation in our study is the increasing incidence of AF‐related ischemic stroke throughout the observation period, in both female and male patients. On the other hand, all‐cause death fell over the same time period. This contrasts with previous studies reporting improving mortality and stroke rates in patients with AF over recent years, likely explainable by improved oral anticoagulation rates.^31^, ^32^, ^33^, ^34^ Indeed, studies have shown gradually improving anticoagulation rates over the past 2 decades, increasing from 37% to 48% of eligible patients with AF in a study from 2008 to 2014 in the United States^32^ and 52% to 74% in a Swedish study from 2012 to 2017.^33^ By including only nonanticoagulated patients with AF in our analysis, our data suggest that these patients may remain at significant risk of ischemic stroke and cardiovascular events, highlighting the need for improved stroke mitigation strategies in this cohort. Of note, the increasing number of nonanticoagulated patients with AF over the observation period is, we suspect, likely a reflection of the increased contribution of cases to the TriNetX database over this time period rather than a true increase in the prevalence of the condition.

### Study Limitations

We acknowledge the limitations of our study, primarily owing to the database‐driven, retrospective, cohort study design. Despite data quality control measures undertaken by TriNetX, diagnostic coding inconsistencies and errors may have occurred, which may have affected the accurate recording of comorbidities and outcome events. Furthermore, due to data privacy restrictions and legal frameworks within TriNetX, the full, patient‐level data set was not available for review or analysis, with analyses limited to those available within the platform itself. For example, despite being able to calculate aggregated counts and prevalence of specific comorbidities within the platform, calculation of individual patient CHA_2_DS_2_‐VASc score was not possible, prohibiting subgroup analysis according to baseline stroke risk.

Our exclusion of those with a prescription for oral anticoagulation in the 12 months after diagnosis may potentially lead to conditioning on the future, impacting event rates: ideally, stroke rates should be assessed in a nonanticoagulated cohort and censored for anticoagulation initiation^35^; unfortunately, the TriNetX platform does not allow for such an analysis. Further, the reasons for lack of anticoagulation are not known; reasons may include low perceived risk of stroke, contraindication to anticoagulation, inability to access anticoagulation, or patient refusal. As a result, our findings may not be generalizable to all cohorts of nonanticoagulated patients. While TriNetX provides some data on race and ethnicity, we chose not to include these variables in our analysis; given the known complexities of racial and ethnic disparities in stroke risk and health care access, incorporating these factors would require a separate, nuanced analysis beyond the scope of our study. Finally, we were not able to adjust for socioeconomic factors such as education level and income, which are known to be associated with clinical outcomes in AF.^36^ Overall, we intended to identify associations between sex and cardiovascular events in AF, and cannot claim these to be causally linked.

### Conclusions

In this global study of >1.2 million nonanticoagulated patients with AF, the association between female sex and ischemic stroke decreased between 2000 and 2019. Despite this, female sex remained associated with a higher stroke risk by the end of the observation period after adjustment for age and cardiovascular comorbidities. Male sex was associated with higher risk of all‐cause death and myocardial infarction, while the risk of dementia and heart failure was similar between sexes. Female sex may therefore remain an important risk modifier in patients with AF.

## Sources of Funding

None.

## Disclosures

D.G. reports institutional research grants from Biosense Webster, Boston Scientific, and Medtronic; and speaker fees from Boston Scientific. G.Y.H.L. is a consultant and speaker for BMS/Pfizer, Boehringer Ingelheim, Daiichi‐Sankyo, and Anthos. No fees are received personally. G.Y.H.L. is a National Institute for Health and Care Research senior investigator and co–principal investigator of the Atrial Fibrillation Integrated Approach in Frail, Multimorbid and Polymedicated Older People project on multimorbidity in AF, which has received funding from the European Union's Horizon 2020 research and innovation program under grant agreement number 899871. The remaining authors have no disclosures to report.

## Supporting information

Data S1
Tables S1‐S2
