# Supplementary material for: Temporal Trends in the Association Between Female Sex and Ischemic Stroke Among Patients With Atrial Fibrillation
Source: J Am Heart Assoc. 2025 Jun 23;14(13):e040325. doi: 10.1161/JAHA.124.040325 (PMC12449936; doi:10.1161/JAHA.124.040325)
Supplement: Supplementary file 1 — Data S1 Tables S1‐S2 [file JAH3-14-e040325-s001.pdf]

Supplemental Material

Supplemental Methods

Data S1: Diagnostic codes

Abbreviations used below:

- *ICD10CM: International Classification of Diseases, Tenth Revision, Clinical Modification*

Diagnostic codes for inclusion, exclusion, and baseline characteristics

Atrial fibrillation:

- ICD10CM:I48: Atrial fibrillation and flutter

Oral anticoagulation:

- RXNORM:11289: Warfarin
- RXNORM:1364430: Apixaban
- RXNORM:1114195: Rivaroxaban
- RXNORM:1599538: Edoxaban
- RXNORM:1037042: Dabigatran

Baseline characteristics:

- ICD10CM:I50: Heart failure
- ICD10CM:I10-11A: Hypertensive diseases
- ICD10CM:E08-E13: Diabetes mellitus
- ICD10CM:I63: Cerebral infarction
- ICD10CM:I21: Acute myocardial infarction
- ICD10CM:I73.9: Peripheral vascular disease, unspecified
- ICD10CM:I70.0: Atherosclerosis of aorta
- ICD10CM:G30: Alzheimer's disease
- ICD10CM:F01: Vascular dementia
- ICD10CM:F03: Unspecified dementia
- ICD10CM:G47.3: Sleep apnea

Diagnostic codes for outcomes

Ischaemic stroke:

- ICD10CM:I63: Cerebral infarction

All-cause mortality:

- "Deceased" code as identified by TriNetX

Myocardial infarction:

- ICD10CM:I21: ICD10CM:I21

Acute heart failure episode:

- ICD10CM:I50.21: Acute systolic (congestive) heart failure
- ICD10CM:I50.31: Acute diastolic (congestive) heart failure
- ICD10CM:I50.811: Acute right heart failure
- ICD10CM:I50.33: Acute on chronic diastolic (congestive) heart failure
- ICD10CM:I50.23: Acute on chronic systolic (congestive) heart failure
- ICD10CM:I50.813: Acute on chronic right heart failure
- ICD10CM:I50.41: Acute combined systolic (congestive) and diastolic (congestive) heart failure

Dementia:

- ICD10CM:G30: Alzheimer's disease
- ICD10CM:F01: Vascular dementia
- ICD10CM:F03: Unspecified dementia

Table S1: Cohort query criteria

Illustration of the cohort query for the identification of female patients from January 1st 2015 to December 31st 2019

**Ungrouped terms**					
must have		demographics	Age	Age (at least 18 years (most recent occurrence))
	and	demographics	UMLS:HL7V3.0:Sex:F	Female
**Group 1**				
**Group 1A**				
must have		diagnosis	UMLS:ICD10CM:I48	Atrial fibrillation and flutter
date constraint		The terms in this group occurred between Jan 1, 2015 and Dec 31, 2019		
event relationship		Any instance of Group 1B occurred within 2 years and 1 day before the first instance of Group 1A		
**Group 1B**				
cannot have		medication	NLM:RXNORM:11289	warfarin
	or	medication	NLM:RXNORM:1364430	apixaban
	or	medication	NLM:RXNORM:1114195	rivaroxaban
	or	medication	NLM:RXNORM:1599538	edoxaban
	or	medication	NLM:RXNORM:1546356	dabigatran
	or	medication	NLM:RXNORM:1037042	dabigatran etexilate
**Group 2**				
**Group 2A**				
must have		diagnosis	UMLS:ICD10CM:I48	Atrial fibrillation and flutter
date constraint		The terms in this group occurred between Jan 1, 2015 and Dec 31, 2019		
event relationship		Any instance of Group 2B occurred within 1 year on or after the first instance of Group 2A		
**Group 2B**				
cannot have		medication	NLM:RXNORM:11289	warfarin
	or	medication	NLM:RXNORM:1364430	apixaban
	or	medication	NLM:RXNORM:1114195	rivaroxaban
	or	medication	NLM:RXNORM:1599538	edoxaban
	or	medication	NLM:RXNORM:1037042	dabigatran etexilate
	or	medication	NLM:RXNORM:1546356	dabigatran
**Group 3**				
**Group 3A**				
must have		diagnosis	UMLS:ICD10CM:I48	Atrial fibrillation and flutter
date constraint		The terms in this group occurred between Jan 1, 2015 and Dec 31, 2019		
event relationship		Any instance of Group 3B occurred at least 1 day before the first instance of Group 3A		
**Group 3B**				
cannot have		diagnosis	UMLS:ICD10CM:I48	Atrial fibrillation and flutter

Table S2: One-year outcomes in adjusted cohorts, comparing females vs. males

	Female			Male			Female vs. male	
	Number at risk (n)	Number with event (n)	**Risk (%)**	Number at risk (n)	Number with event (n)	**Risk (%)**	**Risk ratio (95% CI)**	**P-value**
**Ischaemic stroke**								
2000-2004	2290	40	**1.75**	2290	26	**1.14**	1.54 (0.94-2.51)	0.0826
2005-2009	48881	955	**1.95**	48881	823	**1.68**	1.16 (1.06-1.27)	0.0016
2010-2014	150227	4825	**3.21**	150227	4365	**2.91**	1.11 (1.06-1.15)	<0.0001
2015-2019	297369	12185	**4.01**	297369	11223	**3.77**	1.09 (1.06-1.13)	<0.0001
**All-cause mortality**								
2000-2004	2290	227	**9.91**	2290	265	**11.57**	0.86 (0.72-1.01)	0.0698
2005-2009	48881	4320	**8.84**	48881	5021	**10.27**	0.86 (0.83-0.89)	<0.0001
2010-2014	150227	12244	**8.15**	150227	13958	**9.29**	0.88 (0.86-0.90)	<0.0001
2015-2019	297369	21898	**7.36**	297369	24639	**8.29**	0.89 (0.87-0.90)	<0.0001
**Myocardial infarction**								
2000-2004	2290	51	**2.23**	2290	65	**2.84**	0.79 (0.55-1.12)	0.1880
2005-2009	48881	925	**1.89**	48881	1100	**2.25**	0.84 (0.77-0.92)	<0.0001
2010-2014	150227	3285	**2.19**	150227	4110	**2.74**	0.80 (0.76-0.84)	<0.0001
2015-2019	297369	7836	**2.64**	297369	8924	**3.00**	0.88 (0.85-0.90)	<0.0001
**Heart failure**								
2000-2004	2290	15	**0.66**	2290	14	**0.61**	1.07 (0.52-2.22)	0.8522
2005-2009	48881	682	**1.40**	48881	649	**1.33**	1.05 (0.94-1.17)	0.3624
2010-2014	150227	3862	**2.57**	150227	39951	**2.63**	0.98 (0.94-1.02)	0.3076
2015-2019	297369	12284	**4.13**	297369	12374	**4.16**	0.99 (0.97-1.02)	0.5583
**Dementia**								
2000-2004	2290	39	**1.70**	2290	28	**1.22**	1.39 (0.86-2.26)	0.1758
2005-2009	48881	692	**1.42**	48881	634	**1.30**	1.09 (0.98-1.22)	0.1088
2010-2014	150227	1953	**1.30**	150227	1861	**1.24**	1.05 (0.99-1.12)	0.1338
2015-2019	297369	5372	**1.81**	297369	5212	**1.75**	1.03 (0.99-1.07)	0.1166

Adjusted through propensity-score matching for age, body mass index, and cardiovascular comorbidities: Body mass index; Heart failure; Hypertensive disease; Age; Diabetes mellitus; Stroke (ischaemic); Vascular disease (myocardial infarction, peripheral vascular disease, or atherosclerosis of aorta); Dementia; Sleep apnoea.
